# Hyperosmotic stress enhances cytotoxicity of SMAC mimetics

**DOI:** 10.1038/cddis.2017.355

**Published:** 2017-08-03

**Authors:** Sebastian Bittner, Gertrud Knoll, Martin Ehrenschwender

**Affiliations:** 1 Institute of Clinical Microbiology and Hygiene, University Hospital Regensburg, Franz-Josef-Strauss-Allee 11, Regensburg 93053, Germany

## Abstract

Inhibitors of apoptosis (IAP) proteins contribute to cell death resistance in malignancies and emerged as promising targets in cancer therapy. Currently, small molecules mimicking the IAP-antagonizing activity of endogenous second mitochondria-derived activator of caspases (SMAC) are evaluated in phase 1/2 clinical trials. In cancer cells, SMAC mimetic (SM)-mediated IAP depletion induces tumor necrosis factor (TNF) secretion and simultaneously sensitizes for TNF-induced cell death. However, tumor cells lacking SM-induced autocrine TNF release survive and thus limit therapeutic efficacy. Here, we show that hyperosmotic stress boosts SM cytotoxicity in human and murine cells through hypertonicity-induced upregulation of TNF with subsequent induction of apoptosis and/or necroptosis. Hypertonicity allowed robust TNF-dependent killing in SM-treated human acute lymphoblastic leukemia cells, which under isotonic conditions resisted SM treatment due to poor SM-induced TNF secretion. Mechanistically, hypertonicity-triggered TNF release bypassed the dependency on SM-induced TNF production to execute SM cytotoxicity, effectively reducing the role of SM to TNF-sensitizing, but not necessarily TNF-inducing agents. Perspectively, these findings could extend the clinical application of SM.

Imbalances in pro- and anti-apoptotic proteins disturb cellular death pathways and allow tumor cells to escape from chemotherapy-induced apoptosis. Newer therapeutic approaches aim to reinstate the cell’s death machinery by targeting the pro-survival BCL-2 family members or the cellular inhibitor of apoptosis proteins (IAP) cIAP1, cIAP2 and X-linked IAP (XIAP). Mechanistically, XIAP blocks apoptosis by direct caspase inhibition whereas cIAP1/2 shut down cell death promoting activities of receptor-interacting protein kinase-1 (RIPK1). Overexpression of IAP contributes to the onset of cancer and drug resistance,^1, 2^ which stimulated the development of SMAC mimetics (SM): small IAP-binding molecules mimicking the IAP-antagonizing activity of endogenous second mitochondria-derived activator of caspases (SMAC). SM free caspases from XIAP-mediated inhibition and trigger degradation of cIAP1/2, which is death promoting in two interdependent ways: it initiates autocrine secretion of tumor necrosis factor (TNF) and concomitantly undermines the function of cIAP1/2 to counteract TNF-induced TNF-receptor 1 (TNFR1)-mediated cytotoxicity.^3, 4, 5, 6, 7^ Usually, TNFR1 activation first triggers formation of a receptor-associated signaling complex (termed ‘complex I’). cIAP1/2-mediated ubiquitination of complex I provides a scaffold for recruitment of kinases activating the survival-promoting canonical nuclear factor kappa B (NF*κ*B)-pathway. SM abolish complex I ubiquitination and facilitate its transition to a secondary cytosolic complex (termed ’complex II’), which can induce apoptotic and necroptotic cell death via caspase-8 and RIPK1/RIPK3, respectively.^8^ How SM trigger TNF production is not fully understood, but SM-mediated loss of cIAP1/2 impairs constitutive ubiquitination and subsequent proteasomal degradation of NF*κ*B-interacting kinase (NIK). Increasing NIK levels activate the non-canonical NF*κ*B pathway and transcription of downstream target genes such as TNF.^3, 4, 9, 10^

Efficient killing by SM alone requires both autocrine TNF secretion and TNFR1-mediated cytotoxicity.^3, 4, 5, 6, 7^ Cancer cells that do not release TNF upon SM treatment are consequently resistant even though SM-mediated IAP depletion sensitizes for TNF-induced cell death. Combining SM with exogenously added TNF could overcome this limitation, but is clinically challenging due to the severe cardio-vascular side effects of the cytokine. However, aside from SM-triggered activation of the non-canonical NF*κ*B pathway, a plethora of stimuli can induce cellular TNF production.^11^ Here, we show that hyperosmotic stress boosts SM cytotoxicity in human and murine cells through hypertonicity-induced upregulation of TNF with subsequent initiation of apoptotic and/or necroptotic cell death. Human acute lymphoblastic leukemia (ALL) cells resisted SM treatment under isotonic conditions due to poor SM-induced TNF secretion. Under hypertonic conditions, however, SM treatment displayed a robust, TNF-dependent cytotoxicity. Mechanistically, hypertonicity-triggered TNF secretion bypassed the dependency on SM-triggered TNF production for SM cytotoxicity and reduced the role of SM to TNF-sensitizers, but not necessarily simultaneous TNF-inducers. Perspectively, our findings could extend the clinical application of SM.

## Results

### Hyperosmotic stress and SM synergistically kill L929 cells in a TNF-involving manner

**Figure 1:**
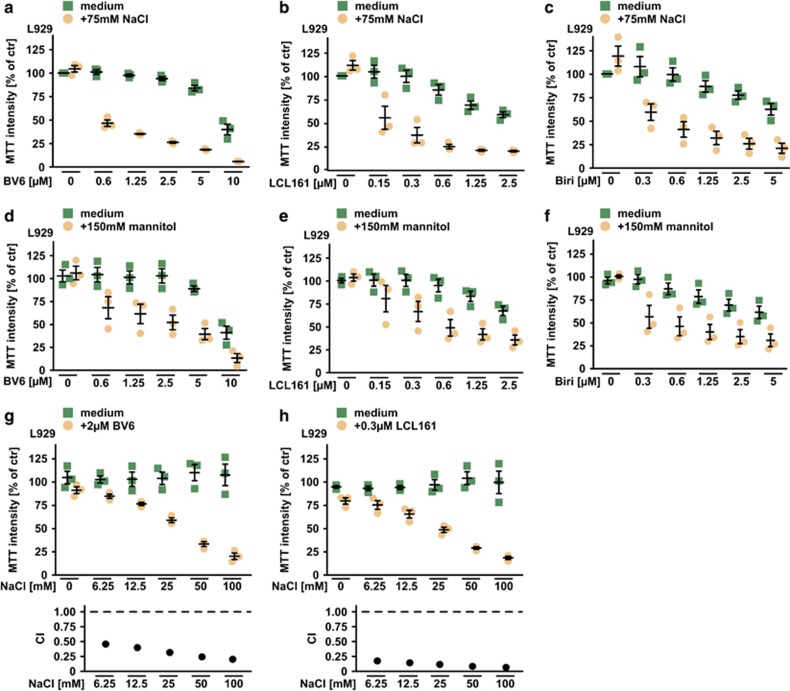
Hyperosmotic stress and SMAC mimetics synergistically kill L929 cells. (**a**–**c**) L929 cells were treated with the indicated concentrations of the SMAC mimetics (**a**) BV6, (**b**) LCL161 and (**c**) birinapant (Biri) in the presence and absence of 75 mM NaCl. (**d**–**f**) Cells were treated as above, but 150 mM mannitol was used instead of NaCl. (**g** and **h**) Top panel: L929 cells were challenged with the indicated concentrations of NaCl in the presence and absence of (**g**) 2 *μ*M BV6 or (**h**) 0.3 *μ*M LCL161. Lower panel: For each combination of SMAC mimetic plus NaCl, the corresponding combination index (CI) value was calculated. CI-values <1 indicate a synergistic effect (see also the Materials and Methods section). Shown are data points and mean±S.E.M. from three independent experiments. For calculation of CI-values, mean values from three independent experiments were used. Biri, birinapant

**Figure 2:**
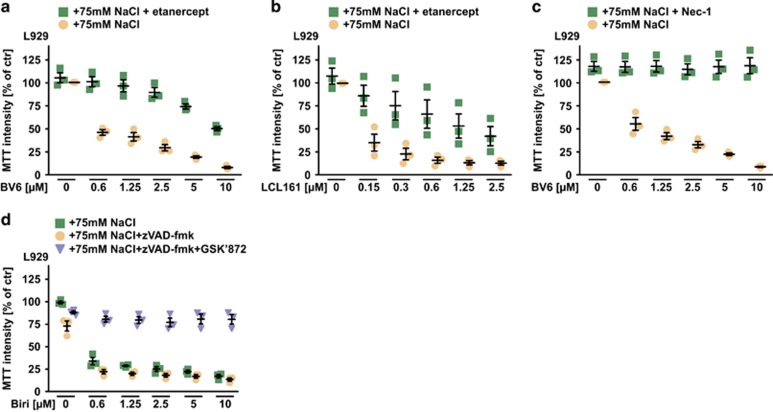
SMAC mimetic-induced cell death under hypertonic conditions involves TNF. (**a** and **b**) L929 cells were grown in medium supplemented with 75 mM NaCl and challenged with the indicated concentrations of (**a**) BV6 and (**b**) LCL161 in the presence and absence of etanercept (400 *μ*g/ml). (**c**) Cells were grown as above and treated with the indicated concentrations of BV6 in the presence and absence of 75 *μ*M necrostatin-1 (Nec-1). (**d**) L929 cells were grown in medium supplemented with 75 mM NaCl and challenged with the indicated concentrations of birinapant in the presence and absence of the pan-caspase inhibitor zVAD-fmk (10 *μ*M) and the RIPK3 inhibitor GSK’872 (2 *μ*M). Shown are data points and mean±S.E.M. from three independent experiments. Biri, birinapant

Addition of osmotically active solutes that cannot diffuse across the cell membrane (e.g. NaCl or mannitol) generates an osmotic pressure gradient between the intra- and extracellular space (hyperosmotic stress or hypertonicity). Hypertonicity generated by supplementing the cell culture medium with 75 mM NaCl or 150 mM mannitol (both increasing osmolarity by 150 mOsm/l) drastically increased cell death in L929 cells upon challenge with the SM BV6, LCL161 and birinapant compared with isotonic conditions (Figures 1a–f). Although NaCl alone displayed no cytotoxic effect up to a concentration of 100 mM, a gradual increase of extracellular NaCl was sufficient to robustly trigger cell death even when low (and under isotonic conditions marginally toxic) concentrations of BV6 and LCL161 were used (Figures 1g and h, upper panel). This pointed to a synergistic effect and was quantitatively assessed by calculating a combination index (CI) for BV6 and LCL161 plus NaCl. Briefly, CI-values <1 indicate a synergistic effect and decreasing CI-values correlate with enhanced synergism of the two compounds.^12^ Synergism in terms of cell death induction was observed for both, BV6 and LCL161 with NaCl (Figures 1g and h, lower panel). Addition of the TNF-neutralizing TNFR2-Fc fusion protein etanercept attenuated, but (especially at high SM concentrations) not fully abrogated SM cytotoxicity in the presence of NaCl (Figures 2a and b). This is in line with the concept that beside TNF-dependent TNFR1 activation^3, 4, 5, 6^ IAP depletion can also kill cells in a TNF-independent, RIPK1-involving manner.^13, 14^ In L929 cells, RIPK1 kinase activity is required for both, SM-triggered TNF release and TNF-mediated cell death.^15, 16, 17, 18^ Pharmacological inhibition of RIPK1 using necrostatin-1 (Nec-1) expectedly abrogated cytotoxicity of BV6/NaCl (Figure 2c). As the pan-caspase inhibitor zVAD-fmk itself induces TNF release and TNF-dependent necroptosis in L929 cells,^15, 19^ birinapant/NaCl cytotoxicity was not reversed by zVAD-fmk alone but required additional GSK’872-mediated inhibition of necroptosis-promoting RIPK3 (Figure 2d). Collectively, these data suggested that hypertonicity enhanced SM-triggered apoptotic and/or necroptotic cell death in a TNF-involving manner.

### Hypertonicity boosts TNF-dependent apoptosis in SMAC mimetic-treated human ALL cells

**Figure 3:**
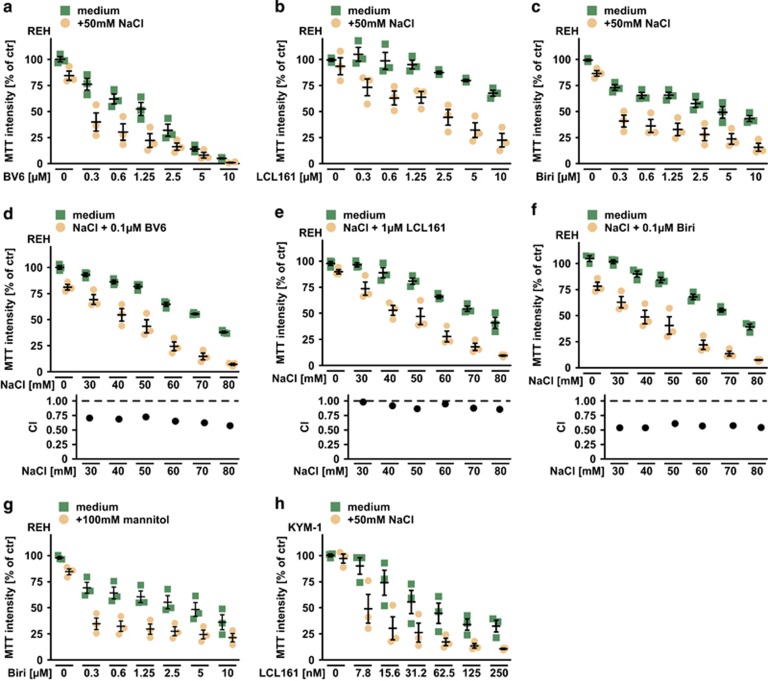
Hypertonicity boosts SMAC mimetic-induced cell death in human ALL cells. (**a**–**c**) REH cells were treated with the indicated concentrations of the SMAC mimetics (**a**) BV6, (**b**) LCL161 and (**c**) birinapant (Biri) in the presence and absence of 50 mM NaCl. (**d**–**f**) Top panel: REH cells were challenged with the indicated concentrations of NaCl in the presence and absence of (**a**) 0.1 *μ*M BV6, (**b**) 1 *μ*M LCL161 or (**c**) 0.1 *μ*M birinapant. Shown are data points and mean±S.E.M. from three independent experiments. Lower panel: For each combination of SMAC mimetic plus NaCl, the corresponding combination index (CI) value was calculated. CI-values <1 indicate a synergistic effect (see also the Materials and Methods section). CI-values were calculated from mean values derived from three independent experiments. (**g**) Cells were treated as in (**c**), but 100 mM mannitol was used to increase osmotic pressure. (**h**) KYM-1 cells were treated as in (**b**). Biri, birinapant

**Figure 4:**
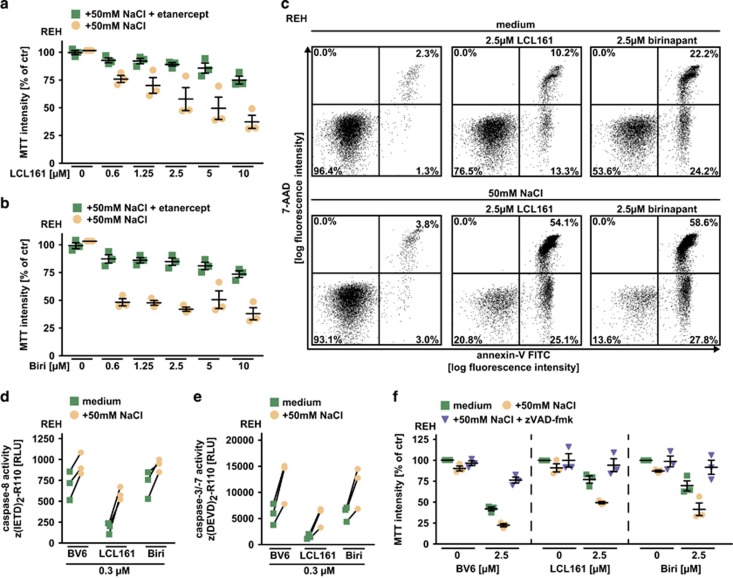
Hyperosmotic stress enhances SMAC mimetic-induced, TNF-mediated apoptosis in ALL cells. (**a** and **b**) REH cells were grown in medium supplemented with 50 mM NaCl and challenged with the indicated concentrations of (**a**) LCL161 and (**b**) birinapant (Biri) in the presence and absence of etanercept (400 *μ*g/ml). Shown are data points and mean±S.E.M. from three independent experiments. (**c**) REH cells were left untreated or challenged with 2.5 *μ*M LCL161 or 2.5 *μ*M birinapant with or without addition of 50 mM NaCl. After incubation for 16 h, cells were analyzed by flow cytometry for positivity of 7-AAD and annexin-V. Data shown are representative of two experiments performed. (**d**and **e**) REH cells were challenged with 0.3 *μ*M BV6, LCL161 and birinapant with or without addition of 50 mM NaCl for 18 h. Caspase-8 and caspase-3/-7 activity was assessed using the fluorogenic substrates (IETD)_2_-R110 and (DEVD)_2_-R110, respectively. Data points from three independent experiments are shown. A line connects caspase activation under isotonic conditions with the corresponding value under hypertonic conditions (50 mM NaCl) for each experiment. (**f**) REH cells were treated with the indicated concentrations of BV6, LCL161 and birinapant with or without addition of 50 mM NaCl and 50 *μ*M zVAD-fmk. Shown are data points and mean±S.E.M. from three independent experiments. Biri, birinapant

Several SM are currently evaluated in clinical trials and recently encouraging results for SM-based therapies in human ALL have been reported.^20, 21^ In human ALL cells (REH) and rhabdomyosarcoma cells (KYM-1), hypertonicity (established by addition of 50 mM NaCl or 100 mM mannitol to the cell culture medium) enhanced BV6-, LCL161- and birinapant-mediated cytotoxicity compared with isotonic controls (Figures 3a–c, g and h). Under isotonic conditions non-toxic concentrations of BV6, LCL161 and birinapant were capable to robustly kill ALL cells when the extracellular osmotic pressure was gradually increased (Figures 3d–f, upper panel). Calculation of the CI revealed values <1, again indicating a synergistic mode of action for SM/NaCl combinations (Figures 3d–f, lower panel). Neutralizing TNF with etanercept significantly diminished SM-related cytotoxicity, demonstrating that (like in L929 cells) TNF was involved in SM-mediated killing of ALL cells facing hyperosmotic stress (Figures 4a and b). Notably, SM-triggered cell death can engage the apoptotic and/or necroptotic pathway,^22^ but apoptosis was apparently the predominant form of cell death in REH cells challenged with SM plus NaCl, as seen by (a) detection of annexin-V and 7-AAD in flow cytometry (Figure 4c); (b) increase of SM-triggered caspase-8 and caspase-3/-7 activation (Figures 4d and e) and (c) almost full-blown rescue with the pan-caspase inhibitor zVAD-fmk (Figure 4f). In sum, these data extended our observations in L929 cells and suggested that hypertonicity and SM acted synergistically in TNF-mediated killing of human ALL cells.

### SM and hypertonicity cooperate in TNF release

**Figure 5:**
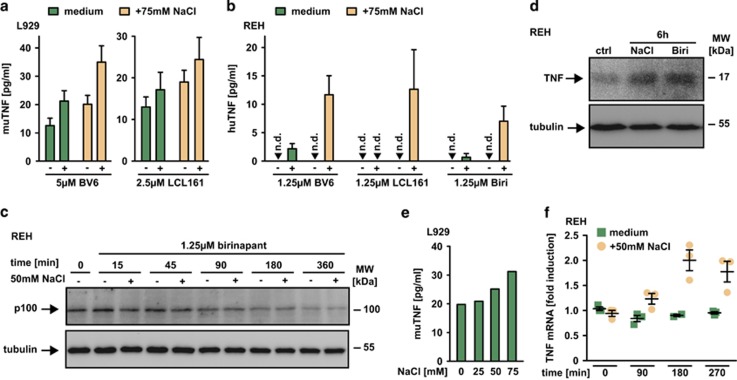
Hypertonicity and SMAC mimetics cooperate in autocrine TNF release. (**a**) L929 cells were cultured with or without addition of 75 mM NaCl and treated with the indicated concentrations of BV6 and LCL161 for 18 h. Supernatants were collected and secretion of murine TNF was quantified using ELISA. (**b**) REH cells were cultured with or without addition of 50 mM NaCl and treated with the indicated concentrations of BV6, LCL161 and birinapant (Biri) for 18 h. Secretion of human TNF in the supernatant was quantified using ELISA. Data shown for (**a**) and (**b**) are mean values±S.E.M. from three independent experiments. (**c**) REH cells were challenged with 1.25 *μ*M birinapant in the presence and absence of 50 mM NaCl for the indicated periods of time. After washing and lysis, western blot analyses were performed with antibodies specific for the indicated proteins. Detection of tubulin served as a loading control. (**d**) REH cells were challenged with 50 mM NaCl or 1.25 *μ*M birinapant (Biri) for 6 h and subsequently analyzed by western Blotting for the presence of the indicated proteins. (**e**) L929 cells were challenged with the indicated concentrations of NaCl for 18 h. Secretion of murine TNF in the supernatant was quantified using ELISA. Data shown are representative of two experiments performed. (**f**) REH cells were challenged with 50 mM NaCl for the indicated periods of time or left untreated. *Tnf* mRNA induction was analyzed by qPCR. Data points of three independent experiments together with mean±S.E.M. are shown. Biri, birinapant

We next evaluated the molecular mechanism underlying SM/NaCl synergism. Apparently, TNF was the key mediator for SM cytotoxicity under hypertonic conditions (Figures 2a, Figures 2b, 4a and 4b). In L929 cells, increase in BV6- and LCL161-induced cell death under hypertonic conditions (Figures 1a and b) was paralleled by enhanced TNF secretion (Figure 5a). Similar observations were made in REH cells, which when challenged under isotonic conditions with low concentrations of SM showed minimal (BV6 and birinapant) or no detectable (LCL161) TNF release (Figure 5b). Again, hypertonicity substantially boosted SM-triggered TNF secretion (Figure 5b) and was accompanied by enhanced SM-mediated cytotoxicity (Figures 3a–c). Mechanistically, this raised the question whether hyperosmotic stress simply increased TNF release initiated by SM via the non-canonical NF*κ*B pathway or itself triggered TNF production. Birinapant-induced non-canonical NF*κ*B activation (as seen by decline of p100) was not enhanced under hypertonic conditions, potentially pointing to an additional, hypertonicity-activated TNF-producing pathway (Figure 5c). This was also supported by experiments that demonstrated (a) increase of TNF levels in REH cells by NaCl and birinapant alone (Figure 5d); (b) NaCl-triggered TNF secretion in L929 cells without SM (Figure 5e) and (c) increased TNF mRNA levels in REH cells cultured under hypertonic conditions (Figure 5f).

### TNF production under hypertonic conditions involves p38 and NFAT5

**Figure 6:**
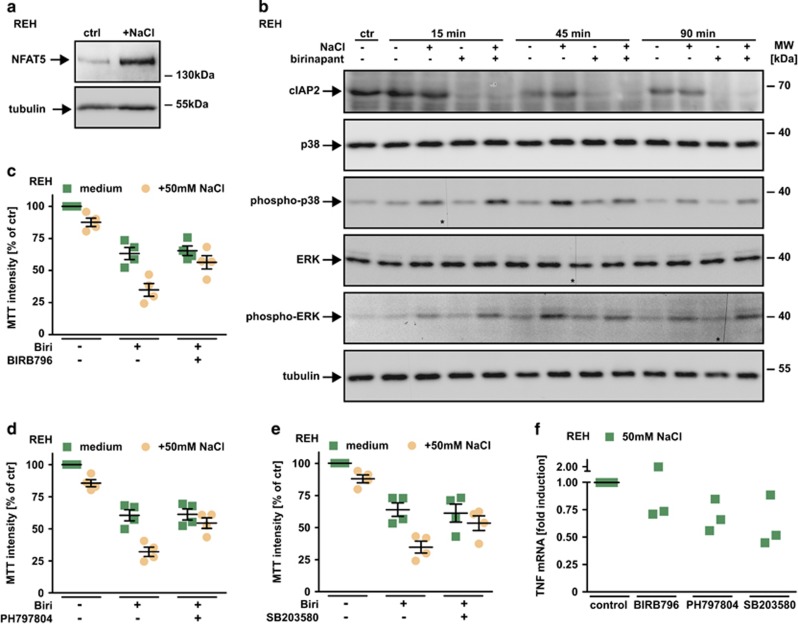
TNF production under hypertonic conditions involves p38 and NFAT5. (**a**) REH cells were cultured in the presence and absence of 50 mM NaCl for 16 h. Cells were washed, lysed and subsequently analyzed using western blotting with antibodies specific for the indicated proteins. (**b**) REH cells were challenged with 1.25 *μ*M birinapant in the presence and absence of 50 mM NaCl for the indicated periods of time. Subsequently, cells were lysed and analyzed by western blot for the indicated proteins. The asterisks (*) in the phospho-p38, ERK and phospho-ERK blots indicate a defect in the CCD sensor of the western Blot imaging system. All samples were run on the same gel, no gels were sliced. (**c**–**e**) REH cells were challenged with 2.5 *μ*M birinapant under isotonic (medium) or hypertonic (+50 mM NaCl) conditions in the presence and absence of the p38 inhibitors BIRB796, PH797804 and SB203580 (5 *μ*M). Shown are data points and mean±S.E.M. from four independent experiments. (**f**) REH cells were challenged with 50 mM NaCl for 210 min in the presence and absence of the p38 inhibitors BIRB796, PH797804 and SB203580 (5 *μ*M). *Tnf* mRNA induction was analyzed by qPCR. Data points of three independent experiments are shown. Biri, birinapant

Upregulation of osmoprotective genes is a vitally important adaptive response to hyperosmotic stress and is critically dependent on the transcription factor NFAT5 (also known as TonEBP) along with its hypertonicity-induced activation through the mitogen-activated protein kinase (MAPK) p38.^23, 24^ Notably, NFAT5 also potently initiates TNF transcription under hyperosmotic stress.^25^ We observed hypertonicity-induced increase in TNF mRNA levels (Figure 5f). The notion that SM cytotoxicity under hypertonic conditions (Figures 1a–c and 3a–c) was associated with higher TNF levels (Figures 5a and b) prompted us to investigate whether the p38/NFAT5 axis could supplement SM-triggered TNF secretion. Expectedly, increased NFAT5 levels (Figure 6a) and phosphorylation of p38 and ERK (Figure 6b) were detectable in REH cells cultured under hypertonic conditions. Birinapant treatment in the presence and absence of NaCl significantly reduced cIAP2 levels, but did not affect p38 and ERK activation. Previous reports demonstrated that p38 inhibition blocked transcriptional activity of NFAT5 and reduced NFAT5-mediated TNF production.^24, 26, 27^ Consequently, pharmacological inhibition of p38 under hypertonic condition should attenuate SM cytotoxicity as TNF upregulation via NFAT5 ceases. Assuming that contribution of both, SM- and hypertonicity-induced TNF production were responsible for SM cytotoxicity, we did not expect a full-blown but partial rescue when blocking only NFAT5-mediated TNF production. Indeed, the p38 inhibitors BIRB796, PH797804 and SB203580 significantly diminished hypertonicity-granted increase in SM cytotoxicity (Figures 6c–e) and (except for one outlier) decreased TNF mRNA levels under hypertonic conditions (Figure 6f). Collectively, these data suggested that triggering autocrine TNF production via hypertonicity-induced p38/NFAT5 activation was underlying synergistic cancer cell killing of SM/NaCl combinations.

### Hypertonicity-induced TNF release overcomes resistance to SM monotherapy

**Figure 7:**
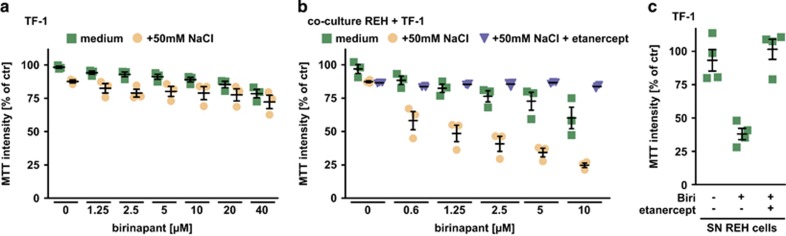
Hypertonicity-induced TNF release overcomes resistance to SM monotherapy. (**a**) TF-1 cells were challenged in the presence and absence of 50 mM NaCl with the indicated concentrations of birinapant. Shown are data points and mean±S.E.M. from three independent experiments. (**b**) Co-cultures of TF-1 and REH cells under iso- and hypertonic (+50 mM NaCl) conditions were challenged with the indicated concentrations of birinapant in the presence and absence of etanercept (1 mg/ml). Shown are data points and mean±S.E.M. from three independent experiments. (**c**) TF-1 and REH cells were challenged with 10 *μ*M birinapant in the presence of 50 mM NaCl for 18 h or left untreated. Supernatant of REH cells was harvested and transferred onto TF-1 cells. After 7 h, viability of TF-1 cells was measured using MTT. Shown are data points and mean±S.E.M. from four independent experiments. Biri, birinapant; SN, supernatant

Thus far, we demonstrated that increased SM/NaCl cytotoxicity is linked to enhanced autocrine TNF signaling (Figures 4a and 5b). As single agents, SM efficacy also depends on autocrine TNF signaling combined with a concomitant ‘death sensitizing’ function. But most tumor entities fail to produce the critical effector cytokine TNF upon SM exposure.^5^ We hypothesized that hypertonicity-derived TNF could compensate for absent autocrine TNF production in SM-resistant cells and reinstate cytotoxicity of SM monotherapies. Indeed, human erythroleukemic TF-1 cells almost completely resisted even high-dose treatments with the SM birinapant (Figure 7a), but SM treatment still sensitized TF-1 cells to TNF generated from nearby cells (paracrine TNF signaling). Challenging co-cultures of ‘SM-resistant’ TF-1 and ‘SM-responsive’ REH cells with birinapant under isotonic conditions revealed a modest degree of cytotoxicity (Figure 7b), ranging expectedly between REH (Figure 3c) and TF-1 cells (Figure 7a) alone. In the presence of 50 mM NaCl, however, cytotoxicity of SM was drastically increased and apparently TNF-dependent, as addition of etanercept almost completely blocked cell death (Figure 7b). This indicates that SM/NaCl-induced TNF release from nearby cells is sufficient to kill SM-sensitized cells that fail to activate autocrine TNF signaling. In line with this, supernatant harvested from SM/NaCl-challenged REH cells efficiently killed SM-sensitized TF-1 cells in a TNF-dependent manner (Figure 7c). Mechanistically, hypertonicity-induced TNF production can substitute in SM-sensitized cancer cells for lack of SM-triggered TNF release.

**Figure 8:**
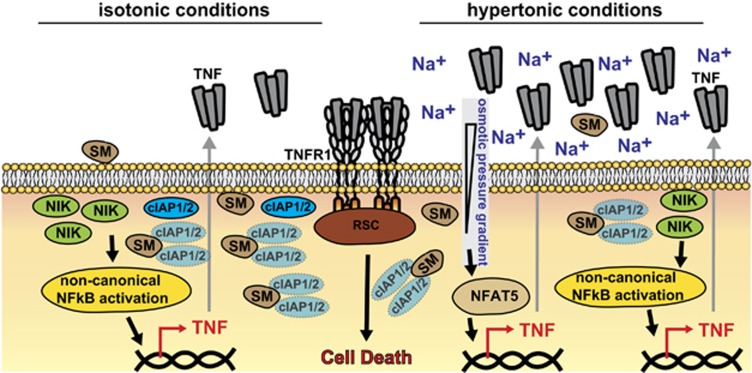
Hypertonicity boosts cytotoxicity of SMAC mimetics. Left side: SM trigger degradation of cIAP and thereby abolishes ubiquitination and subsequent proteasomal degradation of NIK. Increase in NIK levels results in non-canonical activation of the NF*κ*B pathway and transcription of target genes such as TNF. In the absence of cIAP1/2, autocrine TNF secretion induces cell death via TNFR1. *Right side*: Under hypertonic conditions, SM also deplete cIAP1/2 and enable non-canonical NF*κ*B activation, TNF release and TNFR1-induced cell death. Additionally, establishing an osmotic pressure gradient activates NFAT5, a vitally important transcription factor for a cell’s adaptive response to hyperosmotic stress. NFAT5 also triggers TNF transcription, which may restore SM cytotoxicity in cancer cells lacking autocrine SM-induced TNF production. RSC, receptor signaling complex

In sum, we demonstrate that hypertonicity-induced complementary TNF secretion via the p38/NFAT5 axis enhances SM-mediated cell death. Our work provides evidence that activation of alternative endogenous auto- or paracrine TNF sources can attenuate dependency on SM-induced TNF release for efficient cancer cell killing (summarized in Figure 8). Reducing the role of SM to TNF-sensitizing, but not necessarily TNF-inducing agents, could perspectively broaden the clinical application of SM-based therapies.

## Discussion

In cancer cells, SM treatment ideally kills two birds with one stone by simultaneous production of and sensitizing to the effector molecule TNF. In most malignancies, however, SM-induced autocrine TNF production is not functional. Consequently, the response to SM monotherapy in preclinical and clinical studies has thus far been poor.^5, 18^ Nevertheless, SM-mediated IAP depletion lowers the threshold for cell death induction. Clinical trials currently evaluate whether this is exploitable to enhance the anti-tumor activity of standard-of-care cancer therapies.^20^

Alternatively, lack of SM-induced autocrine TNF secretion could be compensated by activating complementary TNF-producing pathways in cancer or other cell types in the tumor environment. For example, TNF is upregulated in response to bacterial and viral pathogen recognition by the innate immune system. Intra-vesical instillation of Bacillus Calmette-Guérin (BCG) to treat early stages of bladder cancer triggered local inflammation and TNF release from recruited neutrophils. In combination with SM, neutrophil-derived TNF potently induced apoptosis.^28^ Additionally, oncolytic viruses synergized with SM in a TNF-dependent manner to promote tumor death in mouse models of breast cancer and glioblastoma.^29, 30^ Cyto- and chemokine upregulation in response to microbial invaders is stringently controlled and usually self-limiting. Thus, innate immune stimuli could clinically be useful to enhance cytotoxicity of SM through induction of a potent yet safe ‘cytokine storm’. However, this approach might be hampered in patients suffering from leukopenia during standard-of-care chemotherapies or in patients treated with immunosuppressive drugs in the course of transplantation.

Notably, exogenous stimuli such as hyperosmotic stress can also initiate endogenous TNF production in cancer cells,^26^ mediated through binding of the transcription factor NFAT5 to the TNF promoter.^25^ Hypertonicity-induced TNF release from cancer cells (and potentially also from cells in the tumor environment) could therefore substitute for lacking SM-induced TNF production, while still relying on the TNF-sensitizing activity of SM. Our study strengthened this concept by showing that hyperosmotic stress (a) initiated TNF release in human and murine cells (Figure 5), (b) enhanced SM-mediated cytotoxicity in a TNF-dependent manner (Figures 1, 2, 3, 4 and 7) and (c) efficiently killed cancer cells in combination with SM even when SM-induced TNF production was poor or absent (Figures 5 and 7). Admittedly, hypertonicity-induced increase in TNF levels might aggravate TNF-related side effects. Cytokine release syndrome, for example, has been reported as dose-limiting toxicity of systemic LCL161 administration in humans and is most likely attributable to SM-induced, NF*κ*B-mediated upregulation of pro-inflammatory cytokines (including TNF).^31^ However, artificially establishing (transient) hypertonic conditions in a narrowly defined area such as the tumor environment could attenuate systemic toxicity by spatially restricting TNF production and lowering SM concentrations necessary for cancer cell elimination (Figures 1g and h and 3d–f). At least in solid tumors, constant release of non-diffusible osmolytes from implantable devices (technically comparable to carmustine-releasing polymer implant wafers in malignant glioma treatment) could establish local osmotic pressure gradients and help to exert maximum SM cytotoxicity to the tumor while reducing damage in adjacent healthy tissues.

Interestingly, our results also highlight a new facet of p38 in SM cytotoxicity. A recent study elegantly demonstrated that inhibition of p38 or its downstream kinase MK2 enhanced the anti-leukemic activity of SM, essentially by increasing SM-mediated TNF production.^32^ Hyperosmotic stress activates p38,^33^ raising the possibility of reduced SM cytotoxicity (due to lower TNF production) under hypertonic conditions. Our results, however, indicate the opposite. Although p38 activation was clearly detectable under hypertonic conditions (Figure 6b), SM treatment resulted in higher TNF levels compared with isotonic controls (Figures 5a and b). At a first glance, this observation seems to contradict the aforementioned study. Under hypertonic conditions, p38-dependent activation of the transcription factor NFAT5 not only upregulates osmoprotective genes^23, 24, 33^ but also TNF.^25^ Several studies reported reduced hypertonicity-induced TNF production upon p38 and/or NFAT5 inhibition.^26, 27, 34^ In line with this, we observed lower *Tnf* mRNA levels and (Figure 6f) reduced SM cytotoxicity under hypertonic conditions in the presence of p38 inhibitors (Figures 6c–e). Whether p38 activation enhances or attenuates SM cytotoxicity might therefore depend on the pathway utilized to supply the key effector molecule TNF. Whereas under isotonic conditions inhibition of p38 boosted TNF secretion by enhancing SM-induced JNK1/2 and ERK1/2 phosphorylation,^32^ p38 inhibition under hypertonic conditions blunted NFAT5 activation and transcription of its target gene TNF.^26, 27, 34^ Activation of an additional endogenous TNF-producing pathway might be of special relevance in tumor cells that respond poorly to SM alone.

In sum, our data revealed that hypertonicity enhanced SM-mediated cell death by complementary TNF secretion. Activation of an alternative TNF source attenuated the dependency on SM-induced TNF release for efficient cancer cell killing (summarized in Figure 8). The tumor environment may therefore not necessarily be an unchangeable adverse determinant in cancer therapy. At least in some cancer entities, physico-chemical modulation of the tumor environment could be exploited to enhance treatment efficacy and broaden clinical application of SM-based therapies.

## Materials and methods

### Cell lines, antibodies and reagents

REH (#ACC-22), TF-1 (#ACC334) and L929 (#ACC-2) cells were obtained from the German Collection of Microorganisms and Cell Cultures (Braunschweig, Germany), KYM-1 cells were kindly provided by Harald Wajant (University of Wuerzburg). All cell lines were grown RPMI 1640 medium (PAN Biotech, Aidenbach, Germany) supplemented with 10% (v/v) fetal calf serum (Sigma, Steinheim, Germany). TF-1 cells were additionally supplemented with 5 ng/ml human granulocyte–macrophage colony-stimulating factor (Immunotools, Friesoythe, Germany). Antibodies used in the study: TNF #3707, p38 #9212, phospho-p38 #9211, ERK #4695, phospho-ERK #4370 (Cell Signaling, Beverly, MA, USA); tubulin #MS-581: Dunnlab (Asbach, Germany); NFAT5 #PA1-023: Thermo Fisher Scientific (Waltham, MA, USA). Chemicals: MTT(3-[4,5-dimethylthiazol-2-yl]-2,5-diphenyl tetrazolium bromide): Biomol (Hamburg, Germany); zVAD-fmk (carbobenzoxy-valyl-alanyl-aspartyl-(Omethyl)-fluoromethylketone): Bachem (Bubendorf, Switzerland); BIRB796, PH797804, SB203580, BV6, LCL161 and birinapant: Selleck Chemicals (Houston, TX, USA). Necrostatin-1 (Nec-1): Stress-Marq (Victoria, Canada); Etanercept was obtained from Pfizer (Berlin, Germany).

### MTT-based cell viability assay

L929 (2 × 10^4^ cells/well), REH cells (7 × 10^4^ cells/well) and TF-1 cells (7 × 10^4^ cells/well) were seeded in 96-well plates and challenged with the indicated concentrations of SM in triplicates (technical replicates). For co-culture assays, 1:1 mixtures of TF-1 and REH cells (5 × 10^4^ cells of each cell line/well) were seeded in 96-well plates and stimulated as above. Cell viability was determined 18 h after stimulation using MTT staining (2 h at 37 °C). Staining intensity was measured at 595 nm and the mean was calculated from the technical replicates of each experiment. The mean value for untreated controls was set to 100%. For any other condition, the MTT staining intensity is given relative to the corresponding untreated group (% of control). Data points shown are mean values (calculated from 3 technical replicates) of independent experiments (*n*≥3).

### Western blot analysis

Cells were harvested, spun down and were directly dissolved in 4 × Laemmli sample buffer (8% (w/v) SDS, 0.1 M dithiothreitol, 40% (v/v) glycerol, 0.2 M Tris, pH 8.0) supplemented with phosphatase inhibitor cocktails-I and -II (Sigma). Samples were sonicated and boiled for 5 min at 96 °C before proteins were separated by SDS-PAGE and transferred to PVDF membranes. To block non-specific binding sites, membranes were incubated in TBS containing 0.1% (v/v) Tween 20 and 5% (w/v) dry milk before primary antibodies of the specificity of interest were added. Antigen–antibody complexes were visualized using horseradish peroxidase-conjugated secondary antibodies (Dako, Hamburg, Germany) and ECL technology (Pierce, Rockford, IL, USA).

### Calculation of CI

CI-values were calculated with the freely available software CompuSyn version 1.0 using the median effect/combination index isobologram method.^12^ In this model, CI-values <1 are considered to be synergistic and CI >1 indicate antagonistic effects. Strength of synergism can be further graded: <0.1 very strong synergism, 0.1–0.3 strong synergism, 0.3–0.7 synergism, 0.7–0.85 moderate synergism, 0.85–0.9 slight synergism, 0.9–1.1 no synergism but nearly additive effects.

### Enzyme-linked immunosorbent assay

For measuring TNF secretion, L929 cells (2 × 10^4^/well) or REH cells (1 × 10^6^/well) were seeded in 96-well tissue culture plates and cultured overnight. The next day, cells were challenged with SM in the presence and absence of NaCl. The supernatant was collected and TNF was quantified using an enzyme-linked immunosorbent assay (BD Biosciences, Heidelberg, Germany). All groups were analyzed as triplicates (technical replicates).

### Caspase activity assays

Caspase activity was measured using the caspase-3/-7 and caspase-8 activity kit (AAT Bioquest, Sunnyvale, CA, USA) according to the manufacturer’s instructions. Emitted fluorescence was quantified using a Victor3 Multilabel Reader (Perkin Elmer, Waltham, MA, USA).

### qPCR

qPCR was essentially performed as described previously.^35^ In brief, total RNA was isolated using the Qiagen RNeasy mini kit (Valencia, CA, USA) according to the manufacturer’s instructions. Two micrograms of total RNA were transcribed into complementary DNA using the high-capacity cDNA reverse transcription kit (Applied Biosystems, Carlsbad, CA, USA). *Tnf* mRNA levels were quantified using the TaqMan human *Tnf* (Hs00174128_m1) gene expression assay (Applied Biosystems) and an ABI Prism 7900 sequence detector (Applied Biosystems). qRT-PCR reactions were performed in duplicates for each sample of an experiment and normalized to the housekeeping gene *Hprt1* (Hs02800695_m1). mRNA levels were calculated using the SDS 2.1 software (Applied Biosystems).

### Flow cytometry

Cell death was assessed by Annexin-V and 7-AAD staining. In brief, REH cells were challenged with 2.5 *μ*M LCL161 and birinapant for 16 h in the presence and absence of 50 mM NaCl. Afterwards, cells were stained with 7-AAD and Annexin-V (4 °C for 15 min in the dark) and analyzed immediately using a FACSCanto flow cytometer (BD Biosciences) following standard procedures.
